# Spontaneous regression of an isolated retinal astrocytic hamartoma in a newborn: a case report

**DOI:** 10.1186/s12886-023-03135-5

**Published:** 2023-09-26

**Authors:** Bo Yang, Danfeng Li, Jun Xiao

**Affiliations:** Department of Ophthalmology, The Second Hospital of Jilin University, No.218, Ziqiang Street, Nanguan District, Changchun City, Jilin Province China

**Keywords:** Retinal astrocytic hamartoma, Spontaneous regression, Newborn

## Abstract

**Background:**

To report the spontaneous regression of an isolated retinal astrocytic hamartoma in a newborn. During the seven-month follow-up duration, fundus photography and fluorescein angiography examinations were performed.

**Case presentation:**

An isolated retinal astrocytic hamartoma was detected in the nasal retina of the left eye of a 4-day-old male infant. At the time of initial presentation, we detected a solitary yellowish-white flat mass with an approximate size of 1.5 disc diameters in the nasal retina. Fluorescein angiography (FA) revealed a diffuse hyperfluorescence with slight fluorescence leakage. Seven months later, the fundus examination showed no lesion in the left eye, FA revealed mild tortuous vessels without leakage.

**Conclusions:**

In the present case, we established that the isolated retinal astrocytic hamartoma in this infant has underwent spontaneous regression. This case can point out that follow –up reexaminations are advisable for a solitary yellowish-white flat mass of the fundus in a newborn.

**Supplementary Information:**

The online version contains supplementary material available at 10.1186/s12886-023-03135-5.

## Background

Retina astrocytic hamartoma (RAH) is a rare benign glial tumor arising from a retinal nerve fiber layer. It is the most common ophthalmoscopic finding in patients with tuberous sclerosis complex (TSC). RAH may also rarely occur in patients with neurofibromatosis type 1 (NF1) or as isolated cases [1]. Few cases of isolated retinal astrocytomas in newborns have been reported in the literature, and spontaneous regression is rare. In this case report, we describe gradual spontaneous regression of an isolated RAH in a newborn.

## Case presentation

**Fig. 1:**
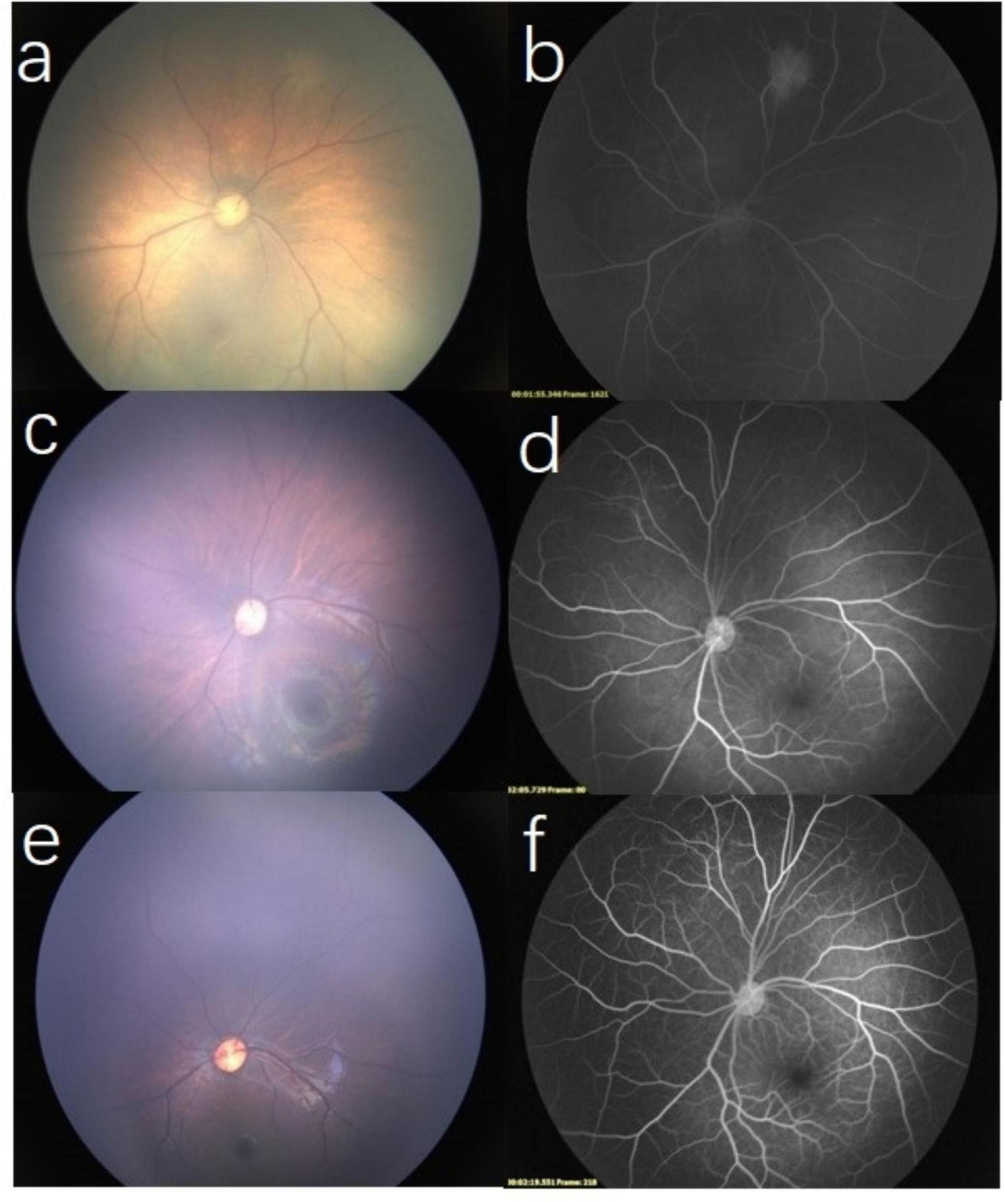
(a) A solitary yellowish-white flat mass in the nasal retina; (b) Fluorescein angiography (FA) revealed a diffuse hyperfluorescence with slight fluorescence leakage.( c) Regression in the size of the retinal mass was observed three months later; (d) FA demonstrated slight mottled hyperfluorescence over the surface of the lesion. (e) The fundus examination indicated no lesion; (f) FA showed mild tortuous vessels without leakage

A 4-day-old male infant, who was born at 40 weeks’ gestational age and weighing 3660 g. He was delivered at term by episiotomy combined with forceps and admitted to the Department of Neonatology of The Second Hospital of Jilin University due to dyspnea in February 2022. No cutaneous lesions were observed. The patient underwent brain magnetic resonance imaging (MRI), the results of which showed a short T1 strip signal shadow (hemorrhage) under the cranial plate of the occipital region on both sides. He had no family history of diseases.The right eye appeared normal in the routine retinal screening performed with RetCam 3 (Clarity Medical Systems, Inc., Pleasanton, CA, USA). However, a color fundus photograph of the left eye revealed a solitary yellowish-white flat mass with an approximate size of 1.5-disc diameters located in the nasal retina (Fig. 1a). Diffuse hyperfluorescence with slight fluorescence leakage was observed by fluorescein angiography (FA) (Fig. 1b). On B-scan ultrasound, the mass was inapparent, with no distinct foci of calcification. No treatment was administered and follow-up reexaminations were recommended. Three months later, regression in the size of the retinal mass was observed (Fig. 1c). FA showed decreased vascularization of the lesion and slight mottled hyperfluorescence over its surface (Fig. 1d). Seven months later, the fundus examination indicated no lesion in the left eye (Fig. 1e). FA showed mild tortuous vessels without leakage (Fig. 1f). The right retina was normal, and thus the clinical course suggested that the isolated RAH may undergo spontaneous regression.

## Discussion and conclusion

Retinal astrocytic hamartoma represents the most common ophthalmoscopic finding in patients with TSC. Based on two relatively large cohorts of patients with TSC, this tumor was detected in 34–36% of patients and, when present, it served as a marker for systemic tumors [2]. Shields et al. [3] found that 81% of patients with classic RAH had no other signs of TSC after systemic evaluation and that only 19% fulfilled TSC criteria. They considered that RAH could be found more commonly as a solitary, independent retinal tumor without clinical or genetic evidence of TSC.

Based on its ophthalmoscopic appearance, RAH is categorized into three types [4]. Type 1 is a subtle, relatively flat, semitransparent lesion located in the retinal nerve fiber layer, most commonly near or at the posterior pole. Type 2 is an easily recognized opaque, white, elevated, and multinodular calcified lesion that is frequently described as resembling a mulberry. These lesions are most commonly located near or at the margin of the disc, although they may also be observed in the midperipheral retina. Type 3 is a transitional type with mixed features of both types 1 and 2 RAH. Our patient was classified as Type 1. The vascularization of the tumor becomes evident from FA images showing numerous abnormal vessels on the tumor’s surface [5]. Type 1 lesions cause a mild blockade of the background fluorescence, hyperfluorescence with leakage from tumor vessels in late-phase [5, 6]. RAH usually remains stable over many years; however, in rare cases, the tumor can enlarge to cause retinal exudation, vitreous hemorrhage, and retinal detachment. Few cases of spontaneous regression of RAH have been reported in the literature. A 12-year-old male with a history of tuberous sclerosis was diagnosed with RAH in the left eye. He was followed up for two years, and the tumor totally regressed by the end of the follow-up period [7]. In another case, gradual spontaneous regression of isolated RAH occurred in an 11-year-old girl [8]. Nevertheless, spontaneous regression of RAH is rare in neonates.

The differential diagnosis of this particular lesion is retinoblastoma in an infant. In a larger series of 604 cases of pseudoretinoblastoma, 15 cases were with diagnoses of RAH. Of them, three were of children who were one year of age or younger in [9]. According to the International Intraocular Retinoblastoma Classification (Murphree) [10]. In a child with neonatal retinoblastoma, the tumor is usually of a higher grade than Group A, typically assigned to Group B, because of the tumor proximity to the foveola or the optic disk rather than because of its size [11]. Thus, it is difficult to distinguish retinoblastoma from retinal astrocytoma in infants. The following characteristics of neonatal retinoblastoma should be considered: (1) a positive family history; (2) unilateral disease at the time of diagnosis is common, but it has a very high rate of becoming bilateral [11]. The patterns of spontaneous retinoblastoma regression were previously classified based on the postirradiation tumor regression pattern as follows: Type 1, with a completely calcified mass with an appearance resembling cottage cheese; Type 2, with a completely noncalcified mass surrounded by RPE changes; Type 3, with a partially calcified mass surrounded by RPE changes; Type 4, with predominant RPE atrophy and focal areas of RPE hyperplasia with minimal overlying translucent tumor; and spontaneous phthisis bulbi [12, 13]. The patterns of retinoblastoma and RAH are completely different. Therefore, in this case, it is not recommended to give laser photocoagulation immediately. Follow-up reexaminations are advisable in such cases.

Based on findings on fundus and fluorescein angiography images, and the process of regression, isolated RAH was a reasonable diagnosis in this patient. This case is a rare example of spontaneous regression of RAH in a newborn. This case can point out that follow –up reexaminations are advisable for a solitary yellowish-white flat mass of the fundus in a newborn.

### Electronic supplementary material

Below is the link to the electronic supplementary material.

Supplementary Material 1

## Data Availability

The datasets used and/or analyzed during the present study are available from the corresponding author on reasonable request.

## Abbreviations

RAH: Retina astrocytic hamartoma
TSC: tuberous sclerosis complex
NF1: neurofibromatosis type 1
MRI: magnetic resonance imaging
FA: fluorescence angiography
