# Supplementary material for: Spontaneous regression of an isolated retinal astrocytic hamartoma in a newborn: a case report
Source: BMC Ophthalmol. 2023 Sep 26;23:395. doi: 10.1186/s12886-023-03135-5 (PMC10523719; doi:10.1186/s12886-023-03135-5)
Supplement: Supplementary file 1 — Supplementary Material 1 [file 12886_2023_3135_MOESM1_ESM.docx]

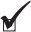

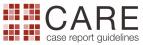

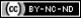
**CARE** **Checklist** **of** **information** **to** **include** **when** **writing** **a** **case** **report**


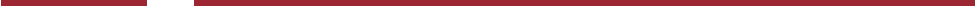


**Item** **Checklist** **item** **description**

**Reported** **on** **Line**

**Topic**


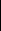


**Title**

**Key** **Words**

**Abstract**

**(no** **references)**

**Introduction** **Patient** **Information**

**Clinical** **Findings**

**Timeline**

**Diagnostic**

**Assessment**

**Therapeutic**

**Intervention**

**Follow-up** **and**

**Outcomes**

**Discussion**

**Patient** **Perspective** **Informed** **Consent**

**1** The diagnosis or intervention of primary focus followed by the words “case report” . . . . . . . . . . . . . . . . . . . . . . . . . . Title，Page1

**2** 2 to 5 key words that identify diagnoses or interventions in this case report, including "case report" . . . Keyword Page 2

**3a** Introduction: What is unique about this case and what does it add to the scientific literature? . . . . . . . . . . . . . . . . Conclusion Page2

**3b** Main symptoms and/or important clinical findings . . . . . . . . . . . . . . . . . . . . . . . . . . . . . . . . . . . . . . . . . . . . . . . . . . . . . Case presentation Page2

**3c** The main diagnoses, therapeutic interventions, and outcomes . . . . . . . . . . . . . . . . . . . . . . . . . . . . . . . . . . . . . . . . . . . Conclusion Page2

**3d** Conclusion—What is the main “take-away” lesson(s) from this case? . . . . . . . . . . . . . . . . . . . . . . . . . . . . . . . . . . . . . Conclusion Page2

**4** One or two paragraphs summarizing why this case is unique (**may** **include** **references**) . . . . . . . . . . . . Background Page3

**5a** De-identified patient specific information. . . . . . . . . . . . . . . . . . . . . . . . . . . . . . . . . . . . . . . . . . . . . . . . . . . . N/A

**5b** Primary concerns and symptoms of the patient. . . . . . . . . . . . . . . . . . . . . . . . . . . . . . . . . . . . . . . . . . . . . . . . . . . .Case presentation Page3

**5c** Medical, family, and psycho-social history including relevant genetic information . . . . . . . . . . . . . . . Case presentation Page3

**5d** Relevant past interventions with outcomes . . . . . . . . . . . . . . . . . . . . . . . . . . . . . . . . . . . . . . . . . . . . . . . . . . . . . . . . N/A

**6** Describe significant physical examination (PE) and important clinical findings. . . . . . . . . . . . . . . . . . .  Case presentation Page3，4

**7** Historical and current information from this episode of care organized as a timeline . . . . . . . . . . . .Case presentation Page3，4.

**8a** Diagnostic testing (such as PE, laboratory testing, imaging, surveys). . . . . . . . . . . . . . . . . . . . . . . . . . . Case presentation Page3，4

**8b** Diagnostic challenges (such as access to testing, financial, or cultural) . . . . . . . . . . . . . . . . . . . . . . . . . . . . . N/A

**8c** Diagnosis (including other diagnoses considered) . . . . . . . . . . . . . . . . . . . . . . . . . . . . . . . . . . . Discussion and Conclusions paragraph 3

**8d** Prognosis (such as staging in oncology) where applicable . . . . . . . . . . . . . . . . . . . . . . . . . . . . . . . . . . . . .  N/A

**9a** Types of therapeutic intervention (such as pharmacologic, surgical, preventive, self-care) . . . . . . . . . . . . . . . .   Case presentation Page3

**9b** Administration of therapeutic intervention (such as dosage, strength, duration) . . . . . . . . . . . . . . . . . . . . . . . . . . . . N/A

**9c** Changes in therapeutic intervention (with rationale) . . . . . . . . . . . . . . . . . . . . . . . . . . . . . . . . . . . . . . . . . . . . . . . . . . . . Case presentation Page 4

**10a** Clinician and patient-assessed outcomes (if available) . . . . . . . . . . . . . . . . . . . . . . .. . . . . . . . . . . . . . . . . . . . . . . . . . . . N/A

**10b** Important follow-up diagnostic and other test results . . . . . . . . . . . . . . . . . . . . . . . . . . . . . . . . . . . . . . . . . . . . . . . . Case presentation Page 4

**10c** Intervention adherence and tolerability (How was this assessed?) . . . . . . . . . . . . . . . . . . . . . . . . . . . . . . . . . . . . . . . . . N/A

**10d** Adverse and unanticipated events . . . . . . . . . . . . . . . . . . . . . . . . . . . . . . . . . . . . . . . . . . . . . . . . . . . . . . . . . . . . . . . . . . . N/A

**11a** A scientific discussion of the strengths AND limitations associated with this case report . . . . . . . . . . . . . . . . . . . . . . . N/A

**11b** Discussion of the relevant medical literature **with** **references**. . . . . . . . . . . . . . . . . . . . . . . . . . . . . . . . Discussion and Conclusions Page 4-6

**11c** The scientific rationale for any conclusions (including assessment of possible causes) . . . . . . . . .Discussion and Conclusions paragraph 2

**11d** The primary “take-away” lessons of this case report (without references) in a one paragraph conclusion . . . . . . Discussion and Conclusions paragraph 4

**12** The patient should share their perspective in one to two paragraphs on the treatment(s) they received . . . . . . . . N/A

**13** Did the patient give informed consent? Please provide if requested . . . . . . . . . . . . . . . . . . . . . . . . . . . . . .  **Yes** ☑ **No**
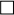


■
